# Biomarkers and clinical predictors of long-term course in obsessivecompulsive disorder: A prospective cohort study

**DOI:** 10.1192/j.eurpsy.2023.533

**Published:** 2023-07-19

**Authors:** S. López-Rodriguez, P. Alonso Ortega, C. Segalàs Cosi, E. Real Barrero, S. Bertolín Triquell, C. Soriano Mas, Á. Carracedo Alvarez, J. M. Menchón Magriña

**Affiliations:** ^1^Obsessive-Compulsive Disorder (OCD) Division, Psychiatry Department, Bellvitge University Hospital, Barcelona; ^2^Medicina Legal y Forense, Universidad de Santiago de Compostela, Santiago de Compostela, Spain

## Abstract

**Introduction:**

The purpose of the research project is to analyze the long-term evolution of obsessive-compulsive disorder (OCD) from of a study of a cohort of patients prospectively followed over a period ranging from 5 to 20 years, treated for according to therapeutic guidelines mediating serotonin reuptake inhibitors (IRS) and drug enhancers (antipsychotics) and cognitive behavioral therapy and evaluated in a standardized manner.

**Objectives:**

To assess the long-term course of Obsessive-Compulsive Disorder (OCD) in a cohort of patients treated according to current clinical guidelines; to analyse possible prognostic factors associated with the long-term course of the disorder including clinical and sociodemographic variables, as well as genetic and neuroimaging biomarkers, and their interaction, and finally to study neuroanatomical and functional cerebral connectivity changes after 15 years of treatment in a subsample of patients.

**Methods:**

Prospective, descriptive, and observational study of a cohort of OCD patients, receiving treatment at the Department of Psychiatry of Hospital de Bellvitge since 1998, according to a standardized protocol. Follow-up period ranges from 5 (n=423), to 10 (n= 247) and 15 years (123). Baseline clinical and sociodemographic assessment, long-term evolution and information on treatments provided are available for the whole sample. Data on whole exome sequencing is available for 300 of the patients included in the cohort and baseline structural neuroimaging and cerebral functional connectivity has been analysed in 168 subjects. To expand the analysis of genetic biomarkers, we propose the study of de novo variants through exome analysis of 50 trios (patient and both parents) selected among those subjects that have reached 15 years of follow-up (25 trios with patients within the “long-term remission” group and 25 trios with patients with chronic OCD). De novo variants detected in the trio analysis will be replicated in the rest of the sample. A structural and resting state MRI will be obtained in a subsample of 100 patients recruited among those who have completed a minimum follow-up period of 15 years, to assess cerebral changes associated with the long-term course of the disorder.

**Results:**

in the current moment the recruitment period of the study has ended and all the data is being statistically analysed in order to provide solid results in a short period of time.

**Conclusions:**

The identification of those factors associated with an increased risk of chronic disease is an element essential to offer personalized treatment to our patients and improve their prognosis, emphasizing the intensive use of those therapeutic strategies for which we can predict a better response and modifying to the extent of, if possible, environmental factors or factors of access to treatment that contribute to perpetuate obsessive symptoms.

**Disclosure of Interest:**

None Declared

